# Compound motion detection based on OAM interferometry

**DOI:** 10.1515/nanoph-2021-0622

**Published:** 2022-02-15

**Authors:** Yuan Ren, Song Qiu, Tong Liu, Zhengliang Liu

**Affiliations:** Department of Basic Course, Space Engineering University, Beijing 101416, China; State Key lab of Laser Propulsion & Application, Space Engineering University, Beijing 101416, China; Department of Aerospace Science and Technology, Space Engineering University, Beijing 101416, China

**Keywords:** compound motion, interferometry, measurement, orbital angular momentum

## Abstract

The simultaneous and independent measurement of multiple movement forms is a significant issue to be solved in research. In this paper, we proposed a method that combines the self-interference of conjugated optical vortices and external interference of plane waves, and successfully realize the independent measurement of both rotation and rectilinear motion. Three kinds of interference schemes based on vortex beams are analyzed theoretically and verified experimentally. The results show that the double interference between conjugated optical vortices and Gaussian beam can detect the motion along and perpendicular to the beam propagation direction even under complex motion background, providing a powerful way to detect the multiple movement forms of a target. Our work may pave a new way for the detection of spatial noncooperative targets and stimulate the invention of new detection equipment.

## 1 Introduction

Objects moving in free space usually possess various forms of movement [1, 2], including but not limited to rotation, rectilinear motion, nutation, vibration, etc. to name a few. It is a universally acknowledged tricky problem to realize the independent measurement of multiple motion parameters of objects [3]. However, it is significant to detect multiple movements simultaneously and independently in many practical applications [4, 5]. The famous Doppler effect provides a powerful tool in detecting moving objects [6] and has been widely used in our daily life, such as Doppler velocimeter [7], color ultrasonic diagnosis [8], et al. But traditional Doppler effect is only sensitive to the linear motion along the beam propagation direction which hence can be called the linear Doppler effect (LDE) [9, 10]. In recent years, with the structured light gradually into people’s vision [11], the light beam with helical phase exp(iℓφ) shows great potential in rotational motion detection [12], [13], [14], [15], where ℓ is an integer expressing the azimuthal quantum number of the vortex light. Each photon in the vortex field carries the orbital angular momentum (OAM) of ℓℏ . When an OAM light illuminates the rough surface of the rotation body along with its axis, the scattered light will Doppler shifted with $\Delta f_{RDE}=\ell \Omega$ , which can be called the rotational Doppler effect (RDE) [16], [17], [18], [19], [20].

Previous researches suggested that both the LDE and the RDE have the same origin but different manifestations [9]. The conventional principle of LDE frequency shift after approximation can be expressed as $\Delta f_{LDE}=f_{0}v\;\cos\;\theta /c$ , where $f_{0}$ is the frequency of the detection light, v means the relative velocity between the wave source and the object, c is the speed of light, θ denotes the angle between v and c [21], [22], [23]. It is obvious that if the motion of the object only exists in the cross-section of the beam propagation, the LDE frequency shift would disappear. That is to say, the traditional plane wave cannot detect the lateral movement which is perpendicular to the beam propagation direction [6, 24].

It is usually to consider the flow of energy in an electromagnetic field in terms of the Poynting vector which can be expressed by S=E×H that has the dimensions of energy per unit time per unit area [25, 26]. The direction of the Poynting vector is always along the propagation direction for a plane wave. But for a beam with a helical phase, there is a skew angle between the Poynting vector and the light propagation direction [27]. This skew angle rotates along the propagation axis and its magnitude can be expressed by α=ℓλ/2πr [28, 29]. In a cylindrical coordinate system, the Poynting vector can be decomposed into two directions, i.e., axial and tangential components [30]. The axial component is supposed to relate to the LDE, while the tangential component is supposed to associate with the RDE [31]. This characteristic shows that the vortex beam has the potential to be sensitive to the motion both in the beam propagation and the perpendicular direction. Significantly, the motion of any tiny particle in space can be decomposed into two orthogonal directions. That is to say, the vortex beam has the potential to detect any kind of motion.

We can decompose the Poynting vector into two orthogonal directions mathematically in a cylindrical coordinate system. However, in practical measurement, the Poynting vector only has one real direction which spirals forward around the vortex beam axis. When measuring the composite moving particles, the scattered light would experience one uniform frequency shift which is the result of LDE shift plus RDE shift [32, 33]. It is difficult to distinguish between the linear and rotational components. Therefore, the multiple forms of motion of the object cannot be measurement independently and simultaneously. To solve this problem, Carmelo et al. proposed the multimode light method, combined with the modulation of the polarization state of the probe beam [34, 35], such that the linear motion and the rotation can be measured separately by superposed optical vortex and the plane wave, respectively. This is an effective method to realize decoupling measurement except its experimental arrangement is a little bit complicated. In addition to this scheme, no other effective decoupling detection method is reported. Especially, the frequency introduced by RDE is usually orders magnitude smaller than the LDE frequency shift in practical application. It is difficult to accurately distinguish a small frequency from a large frequency signal, let alone under the complex motion background.

No matter traditional LDE introduced by plane wave or RDE introduced by optical vortex cannot effectively achieve the simultaneous, independent, and decoupled measurement of multi-movement forms. To overcome the shortcomings of a single beam only sensitive to the motion in one specific direction, we combined the multimode beams through self-interference and external interference based on an OAM interferometry. Both the LDE and the RDE frequency shift can be obtained at a one-time measurement.

## 2 Methods and materials

### 2.1 Interpretation of Doppler effect through interference

In polar coordinates, the electric field E of a monochromatic beam can be expressed in the following form [36],

(1) $$E\left(r,t\right)=\text{ℛ}\left\{A\left(r\right)e^{-i\omega t}\right\}=\frac{1}{2}\left[A\left(r\right)e^{-i\omega t}+A{\left(r\right)}^{*}e^{-i\omega t}\right]$$

where A(r) is a complex vector that possesses three components in the Cartesian coordinate system. When the beam propagates in the uniform medium, we can take $〈E^{2}〉$ to express the magnitude of the light intensity. Suppose there are two beams meet at point P in space, then the total electric field is $E=E_{1}+E_{2}$ , therefore its intensity can be calculated by $E^{2}={\left|E_{1}\right|}^{2}+{\left|E_{2}\right|}^{2}+2E_{1}E_{2}$ . If we take I to represent the scalar intensity, i.e. $I=〈E^{2}〉$ , the light intensity of point P can be written as $I_{P}=I_{1}+I_{2}+J_{12}$ , where $I_{1}$ and $I_{2}$ are the intensity of two single beams respectively, $J_{12}=2\sqrt{I_{1}I_{2}}\cos\;\delta$ is the interference term of the two beams which is the most important factor that determine the distribution of the interference field.

Generally, there is a phase difference between two coherent beams when meeting at point P . This phase difference can be expressed by $\delta =\left(2\pi /\lambda_{0}\right)\cdot \Delta S$ , with ΔS denoting the optical path difference between two beams, $\lambda_{0}$ is the wavelength. Obviously, the total light intensity $I_{P}$ would vary between $-2\sqrt{I_{1}I_{2}}$ and $2\sqrt{I_{1}I_{2}}$ with the light path difference. If the optical path of the two beams does not change with time, stable interference fringes will be generated. While if the optical path difference changes uniformly with time, the interference fringes will move periodically. If a photodetector is placed at point P , both the displacement and velocity can be obtained by recording the change of the fringes. Based on this principle, various interferometers were invented and become the most accurate spatial distance measurement tool.

For a vortex beam that has a helical phase, its electric field can be expressed by [37],

(2) $$E_{OV}\left(r,\varphi ,z\right)=E_{0}\;\exp\left(i\mathrm{kz}\right)\exp\left(i\ell \varphi \right)\exp\left(i\sigma \varphi \right)$$

where ℓ is the azimuthal quantum number or topological charge. σ defines the polarization state, σ=±1 for the right- and left-handed circularly polarized light, and 0 for linearly polarized light. k=2π/λ is the wavevector.

It can be seen from Eq. (2) that the phase of each point in a vortex field is not only related to the propagation distance, also associated with the azimuthal position. The electric field of a plane wave can be expressed as $E_{p}\left(r,\varphi ,z\right)=E_{1}\;\exp\left(ikz_{0}\right)$ . According to the superposition principle of light, when a plane wave interferes with the single-mode optical vortex, the final intensity is,

(3) $$I_{OV-P}={\left(E_{OV}+E_{P}\right)}^{2}={\left|E_{0}\right|}^{2}+{\left|E_{1}\right|}^{2}+2E_{0}E_{1}$$

The third term of the above equation is the interference term which determines the distribution of the interference field. Combined with Eq. (2), the interference term can be present by,

(4) $$I_{cross}=2E_{0}E_{1}\;\exp\left(ik\Delta z+i\left(\ell +\sigma \right)\Delta \varphi \right)$$

where $\Delta z=z-z_{0}$ denotes the optical path difference between two beams along the propagation direction. Δφ denotes the angular phase difference between two beams.

It is worth noting the origin of the azimuthal phase difference Δφ . Since the angular phase is uniform for a plane wave, the azimuthal phase difference comes from the optical vortex itself. The first condition is that if the beam rotates with time, for example, passing through a rotating Dove lens, causing the angular phase change is $\Delta \varphi =\varphi_{0}+\Omega t$ , with $\varphi_{0}$ being the initial phase difference and Ω being the rotating speed of the Dove lens. This phase change Δφ will influence the interference field and cause the fringes to tackle periodically, which is the angular Doppler frequency shift comes from [16, 38, 39]. The second condition is that when the beam illuminates a rotating target, taking the direction of the Poynting vector into consideration, the phase change of the scattered vortex light is $\Omega^{\prime }t$ , where $\Omega^{\prime }$ is the rotational speed of the object. This phase difference will also influence the interference field and modulate the light intensity, which would produce the rotational Doppler frequency shift and also can be understood as rotational Doppler effect [12]. Except for the above conditions, the polarization state also can introduce a frequency shift which can be called the vectorial Doppler effect [40]. But for the vortex field with a uniform polarization state, the azimuthal phase will not be affected by the polarization state.

**Figure 1:**
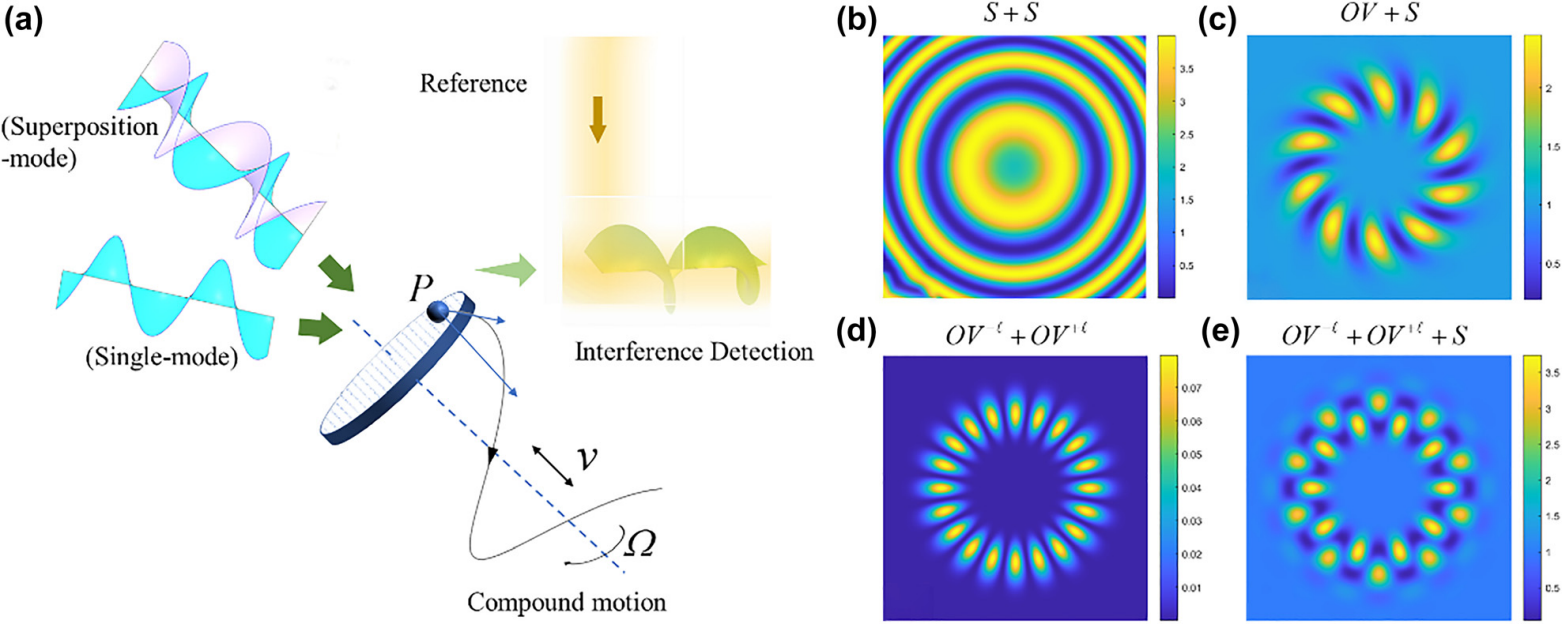
Multimode beam interference scheme and corresponding results. (a) The concept of the OAM based interferometry for the detection of compound motion. (b)–(e) is the simulated interference patterns between multimode beams. (b) Interference between two spherical waves under fully coaxial conditions. (c) Interference pattern generated by single mode optical vortex ( ℓ=10 ) and spherical waves. (d) Self-interference conjugated optical vortices ( ℓ=+10 & ℓ=-10 ). (e) Interference result between conjugated optical vortex ℓ=±10 and spherical wave.

In an OAM based interferometry, as shown in Figure 1(a), the interference fringes would arise at where the detection and the reference light converge. Both the single- and superposition-mode optical vortices can be used as the detection light. The variation characteristics of the interference pattern are defined by the phase difference between the two beams and can be expressed by Eq. (4). For a detection scheme of a practical with compound motion, the phase difference Δz is determined by the linear motion of the targets which can be expressed as $\Delta z=v_{0}t$ . The angular phase difference can be expressed as (ℓ±σ)Ωt which is associated with the angular motion of the targets. If the interference beam is recorded by a photodetector, then only the scaler field intensity is left which can be calculated by taking the absolute value of $I_{cross}$ ,

(5) $$I_{cross}^{\ell -0}\left(t\right)=2\sqrt{I_{0}I_{1}}\cos\left(\frac{2\pi }{\lambda }\cdot 2v_{0}t+2\pi \left(\ell +\sigma \right)\Omega t\right)$$

where Ω is the rotational speed of the target. Therefore, the light intensity change frequency is $f_{L}^{∥}=2v_{0}/\lambda$ for a linearly moving target, and $f_{R}^{⊥}=\left(\ell +\sigma \right)\Omega$ for a rotating target, and $f_{\mathrm{mod}}=f_{L}^{∥}+f_{R}^{⊥}$ is the total Doppler frequency shift for an object moving forward in a spiral trace. When the optical vortex interacts with the moving object, it would arouse an LDE frequency shift the same as the traditional plane wave. The difference is mainly concentrated on the rotational part, only the optical vortex is sensitive to the motion perpendicular to the light propagation direction. Moreover, the total frequency shift is the coupling result of the LDE and RDE shift, which means the linear and angular motion cannot be measured separately by this method.

Using the same superposition principle shown in Eq. (3), we can analyze the interference result between two optical vortices with conjugated topological charge too. There are two ways to realize the interference between two optical vortices. One is the conventional way to split one optical vortex into two and then converge somewhere again; the other method is interference two optical vortices by the spatial light modulator (SLM). For the former method, the Doppler shift would be the same as expressed by Eq. (5), but the interference pattern differs from the optical vortex with the plane wave, as shown in Figure 1(d). For the latter method, if the two optical vortices with conjugated topological charge interference through the SLM, the interference light illuminates from the SLM can be seen as one light with the same polarization state. No matter how far away from the detector or whatever the motion of the object along the propagation direction, the propagation distance would be the same, i.e., Δz=0 . Therefore, the LDE cannot be present by the superposition beam. However, because of the chirality of the optical vortex, the RDE shift would be exactly contrary. Therefore, the intensity after detecting the moving object is,

(6) $$I_{cross}^{-\ell -+\ell }\left(t\right)=2\sqrt{I_{LG1}I_{LG2}}\cos\left(2\pi *2\ell \Omega t\right)$$

Only the rotational motion can be measured through the SLM-based interference vortex light. In our method, this kind of interference is defined as self-interference superposition.

The above interference scheme can obtain the coupling frequency shift and the rotation frequency shift, respectively. This inspires us the new possibility that through the combination of these two schemes, the rotation and the linear motion can be measured simultaneously and independently. In theory, every two beams that satisfy the coherence condition when converging will produce an interference phenomenon as Figure 1 shows. If we mix the plane wave, left-handed and right-handed optical vortices, as analyzed before, the plane wave interference with left-handed vortex will produce a frequency shift of $f_{\mathrm{mod}}^{1}=f_{L}^{∥}+f_{R}^{⊥}$ , while the plane wave interference with right-handed vortex will produce a frequency shift of $f_{\mathrm{mod}}^{2}=f_{L}^{∥}-f_{R}^{⊥}$ . (Detail derivation in Supplement 1) Note that these two frequency shifts are symmetry with the linear frequency shift $f_{L}^{∥}$ on the spectrum. Then the linear motion can be confirmed by the mean value in the spectrum. The third one is the interference between the two self-interference superposition vortex light which will introduce the frequency shift of $f_{\mathrm{mod}}^{3}=f_{R}^{⊥}$ , which can be recognized on the frequency spectrum directly. Up to now, through the OAM beam self-interference and external interference, it becomes a reality to measure the linear motion and rotational motion of the target at the same time.

### 2.2 Experimental methods

**Figure 2:**
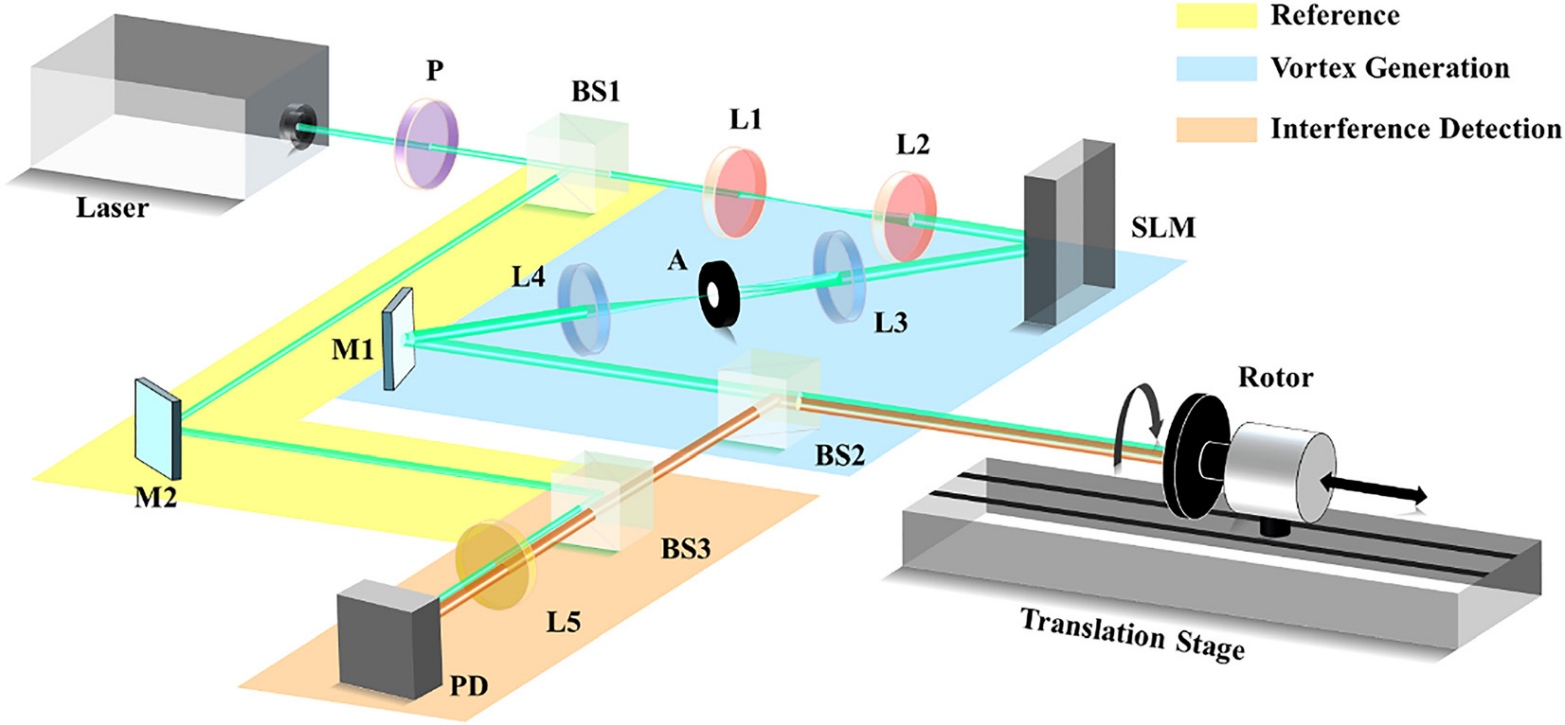
Laboratory experiments on the speed measurement of complex motion. Mainly composited with three branches: Reference, vortex generation, and interference detection. P: polarizer; BS: beam splitter; L: lens; M: mirror; SLM: spatial light modulator. The rotor is arranged on the translation stage to realize rotation and rectilinear motion simultaneously.

In the proof of principle experiment, we designed the interference experimental arrangement. As shown in Figure 2, all the optical path is presented and the circuit part is not listed (Fully experimental scheme see Supplement 1). The interference of the three beams is carried out in two steps. The interference of the superposition vortex light is used as the probe beam, and the self-interference is realized by loading the superposition phase hologram on the SLM. The Gaussian beam emitted from the light source is used as the reference beam, and the superimposed vortex beam scattered back from the target on the surface of the photodetector performs the second time interference. The laser source produces a continuous light wave with a wavelength of 532 nm, the coherence length is 50 m which determines the longest detection range. A linear polarizer is set to obtain the pure horizontal polarized light to match the diffraction direction of the spatial light modulator (SLM). On the other hand, the uniform polarization state of the light can guarantee a good coherence of the light. The lens L1 and L2 are used to expand and collimate the laser to match the size of the SLM LCD screen.

By uploading the LG mode holograms on the SLM, we can generate LG mode optical vortex in any topological charge. The phase mask is designed by the complex amplitude modulation method, which combines both the phase and the amplitude information of the desired optical vortex. Therefore, the optical vortex can keep a good mode purity. Since the value and the power of the rotational Doppler frequency signals are closely related to the mode purity of the incident light. It is essential to guarantee the high mode purity of the detecting vortex beam as much as possible. A 4*f* spatial filter composited by lens L3, L4, and aperture A is used to filter the first-order light. Any kind of light can be generated through the vortex generation section as shown in Figure 2. If a multi-mode light phase mask is uploaded on the SLM, then the SLM would perform as both the modulator and the interferer. The light reflected from the SLM can be well interfered as designed and can be seen as one multi-mode light. This characteristic of the reflected light can be understood as self-inference light. Most significantly, no matter the beam propagates any distance, the optical paths among these modes are the same. Here, the conjugated superposition optical vortex directly illuminates the target to be measured after passing through BS2.

The polarized Gaussian mode laser reflected by BS1 is set as reference light. And in the detection experiment, the reference would converge with the experiment light as BS3. The interference detection section is composited with BS3, L5, and a photodetector (PD). The varying of the interference light can be captured by the PD directly and then connect with the data acquisition card for sampling. The target to be measured is a rotor arranged on a translation stage, which can move linearly and angularly. To obtain a good signal-to-noise ratio, the scattered light should be as strong as possible. So, we paste the metal reflective material on the surface of the rotating plate. On the other hand, the surface of the target cannot be a completely smooth reflecting surface, otherwise, the rotational Doppler frequency shift will lose.

## 3 Results and discussion

**Figure 3:**
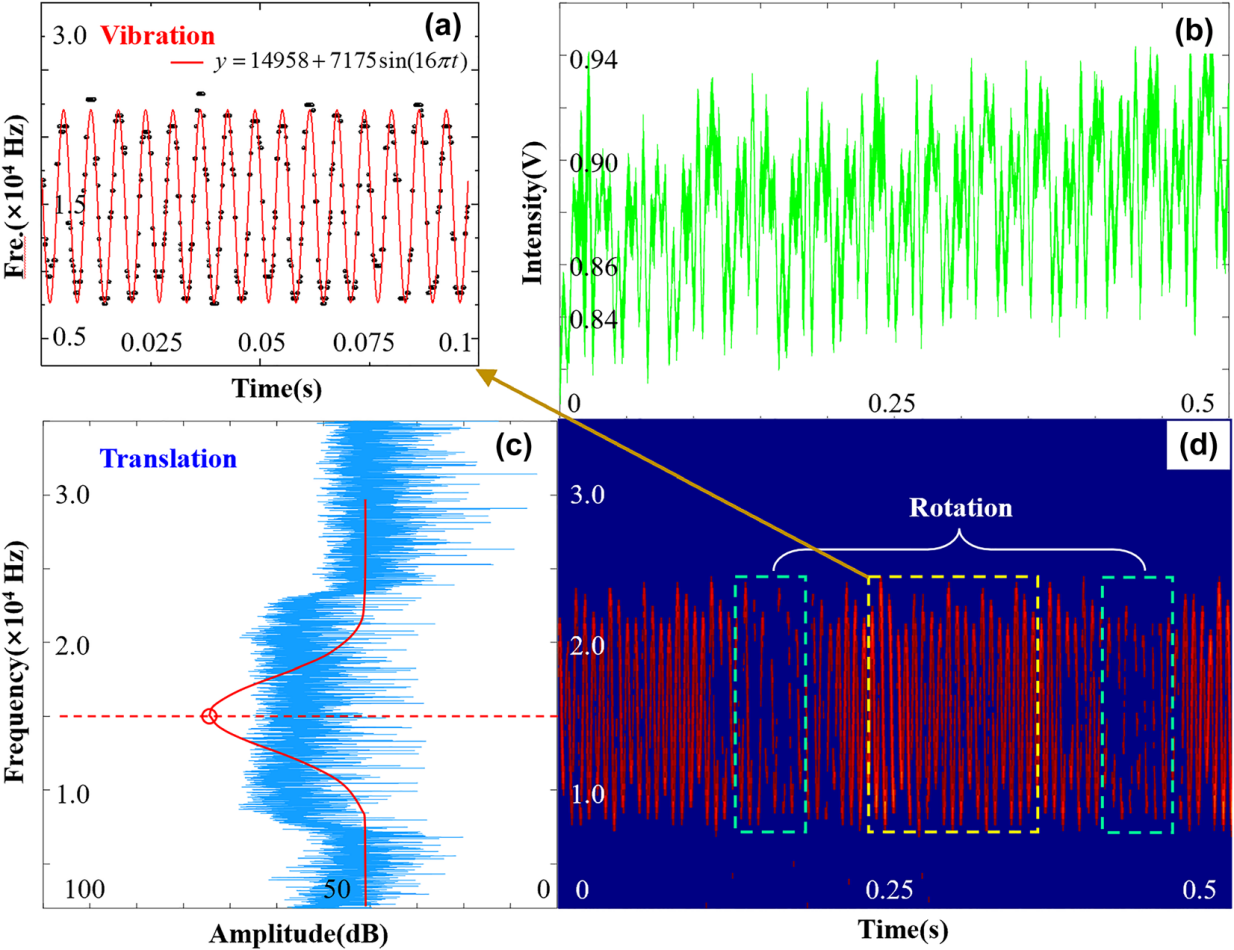
Detection result by the interference of two sphere waves. The speed of the object is set $v_{0}=4.00\;mm/s$ and Ω=3.611 rps . (a) The fitting signal of the local details of the vibration of the stage. The fitting equation manifests both the LDE frequency shift and the vibration frequency. (b) Data captured by the photodetector in the time domain within 0.5 s. (c) The frequency spectrum after Fourier transformation of (b). The red line is the Gaussian fitting result of the broadened signal which gives the value of the LDE frequency shift. (c) The STFT result of the signal. The changes in signal strength are caused by the rotation and may reflect the rotation speed.

Since the experiment is based on the interference phenomenon of the light beam, the accuracy requirements for the experimental device are pretty high. Especially, the small vibration of the rotor and the translating stage would significantly affect the detection results of the system. We first conduct the control experiment to characterize the performance of the experimental setup by the fundamental Gaussian beam. A phase mask of the Gaussian beam is uploaded on the SLM, the reflected beam from SLM is in Gaussian mode correspondingly. Here, the SLM plays a role like a mirror. The whole system can be seen as a modified Michelson interferometer. After a short distance propagation, the reflected beam becomes an approximate spherical wave and therefore would generate ring shape interference fringe. The interference pattern is shown in Figure 1(a). We set the rotational and the translational speed of the object of $v_{0}=4.00\;mm/s$ and Ω=3.611 rps , respectively. The experimental result is investigated both in time-, frequency-, and time-frequency- domain, as shown in Figure 3.

**Figure 4:**
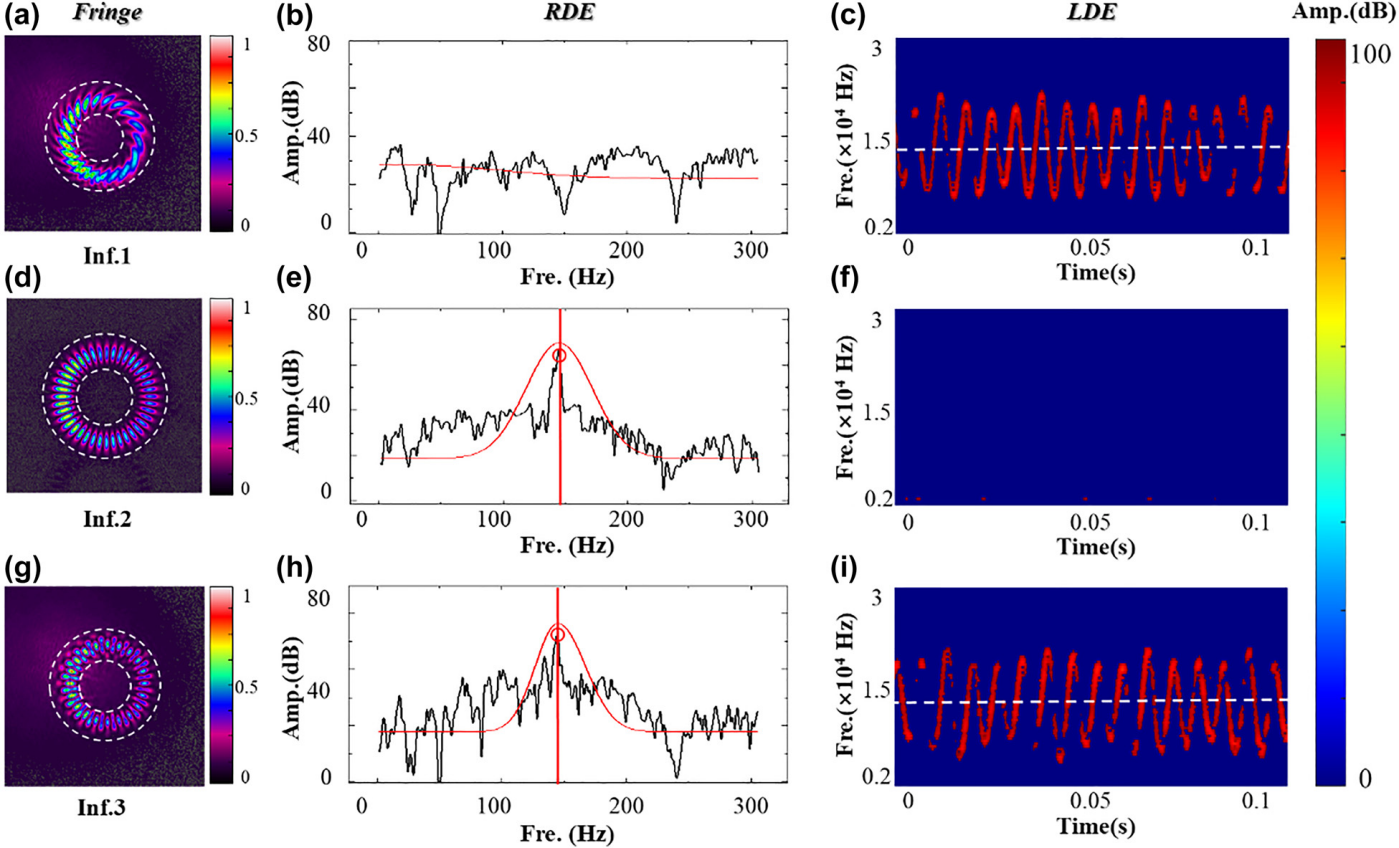
Detection result upon three different interference schemes. Upon all the three measurements, the speed of the object is set rectilinear motion $v_{0}=4.00\;mm/s$ and rotation Ω=3.611 rps . (a)–(c), (d)–(f), and (g)–(i) are the results under vortex–spherical wave external interference (Inf.1), vortex–vortex self-interference (Inf.2), and conjugated vortex–spherical interference (Inf.3), respectively. The RDE frequency shift is obtained from the frequency spectrum in the low-frequency domain as shown in (b), (e), and (h). The LDE frequency shift is calculated by the mean value of the STFT frequency map from (c), (f), and (i).

Figure 3(d) is the time-domain data collected by the photodetector directly within 0.5 s. After a Fourier transformation, the frequency domain signal is presented in Figure 3(c). A clear and broadened frequency signal emerges around the calculated LDE frequency signal. Theoretically, the LDE frequency shift would present in the form of one single peak. After the Gaussian fitting of the frequency spectrum, the center frequency is $1.496\times 10^{4}\;Hz$ which is very close to the participated value of $1.503\times 10^{4}\;Hz$ . The relative error of the measured frequency is 0.4% . To investigate the reason for the broadened frequency signal, we further conduct the short-time Fourier transformation (STFT) of the origin data. From the time-frequency map shown in Figure 3(d), it is clear that the frequency signal fluctuates around the center frequency which is caused by the vibration of the translation stage. Figure 4(a) shows the fitting signal of the vibration. From the fitting equation, both the center frequency and the vibration frequency can be obtained.

Moreover, since the phase of the spherical wave has no difference in the azimuthal direction, only the frequency shift introduced by the rectilinear motion will be left in the detection. But from the STFT map, we can observe that the signal becomes strong from time to time, and the variation frequency is exactly the same as the rotational frequency which manifests that the rotation-sensitive ability. Actually, it is a great challenge to make the reflected light completely coincident with the reference light. If scattered light interferes well with the reference beam, the amplitude of the detected signal becomes strong. Therefore, the rotation information can be seen in the STFT diagram, and this signal strength change has nothing to do with RDE. Up to now, all the confounding factors are analyzed.

After characterizing the moving platform, we conduct the following experiments. We first demonstrate the detection results by the signal mode OV with the topological charge of ℓ=20 . The frequency shift is detected by interference between the scattered light and the plane wave in the interference section. The interference pattern is shown in Figure 4(a), as the object moves along the beam axis, the spiral fringe will rotate associated with the moving distance. This phenomenon differs from the traditional interferometers and can be used to precisely sense the displacement [41, 42]. The motion of the target is set in both rotation and translation with the speed of $v_{0}=4.00\;mm/s$ and Ω=3.611 rps , respectively. According to Eq. (5), there is only one frequency signal which is the combination of the LDE and the RDE, $f_{\mathrm{mod}}=f_{L}^{∥}+f_{R}^{⊥}$ . From Figure 3(c) we can see that there is no frequency signal in the low-frequency domain, while in the high-frequency band where the LDE frequency shift lies clearly shows a frequency signal. The signal frequency is 1.503 × 10^4^ Hz.

Subsequently, we conduct the detection by the self-interference vortex beam. In this experiment, the reference section is blocked. The conjugated superposition vortex phase mask with the topological charge of ℓ=±20 is uploaded on the SLM. The corresponding interference pattern is shown in Figure 4 (d). Compared with the single-mode optical vortex interference, there is an obvious signal in the low-frequency area with the frequency of $f_{R}^{⊥}=145\;Hz$ . The detecting result is very close to the theoretical RED value of 144.4 Hz upon the rotational speed of Ω=3.611 rps . However, there are no signals in the high-frequency domain. The detection results further verify the theoretical analysis that there is no light path difference between the conjugated optical vortices, therefore the superposition vortex is immune to the linear motion.

By introducing the reference beam, we conduct the third time experiment. The object keeps the combined motion of linear and rotation, with the given translation speed of $v_{0}=4.00\;mm/s$ and rotation speed Ω=3.611 rps . This time we use the vortex generation section to produce a self-interference conjugated OAM beam with ℓ=±20 , and then interference with the spherical wave in the interference detection section. The interference pattern is shown in Figure 4(g) which is different from the conventional interference pattern after the double interference. After Fourier transformation of the time domain data, the Doppler frequency signal appears both in the low and high-frequency domain which corresponds to the RDE and the LDE frequency shift, respectively. The RDE shift is obtained after the Gaussian fitting of the frequency spectrum, as shown in Figure 4(h), a clear peak with the frequency of 145.3 Hz shows up in the spectrum. The corresponding measured rotation speed is 3.632 rps with a relative error of 0.6%, shows a very high measurement accuracy. The LDE frequency shift is again obtained by taking the mean value of the frequency over time. In this measurement, the mean frequency shift is $1.498\times 10^{4}\;Hz$ , and the calculated translation speed is 3.984 mm/s , accompanied by the relative error of 0.38%. By employing the self-interference and heterodyne interference, the rotational speed and the linear speed can be measured simultaneously and independently.

**Figure 5:**
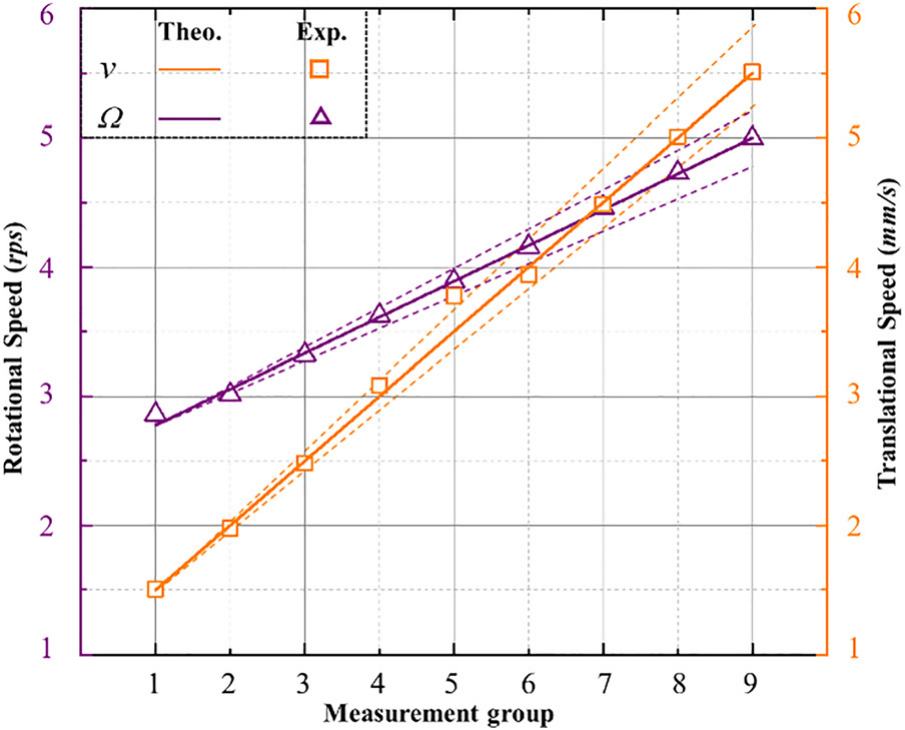
Dependent detection results under different rotation and rectilinear motion speed. The purple line and orange line are the theoretical values. The dot lines give the error range of the measurement results.

To further verify the ability of the method, we measured the compound movement of the object at different speeds. Still, the measurements are conducted upon the reference detection condition of the conjugated optical vortex as the detection light and the Gaussian mode beam as the reference light. The experimental results are shown in Figure 5. For nine times dependent detection with different rotational and rectilinear speeds, the measured result is very close to the theoretical value for every single detection. (The measured values of LDE and RDE frequency can be found in Supplement 1) In all the measurements, there are small differences between theoretical and experimental, as all measurements are subject to some uncertainty due to the limited accuracy of the speed control. But all the relative rotational speed measurement errors are less than 2.9% and all the translational speed measurement errors are less than 2.8%.

It is worth to note that the LDE frequency shift is obtained by the mean value of the frequency shift over time. The scientific nature of this approach lies in two aspects. The first is that the frequency shift introduced by the vibration fluctuates around the central frequency shift, i.e., the linear Doppler frequency. Secondly, the other frequency shift of conjugated optical vortex introduced by the rotation motion is $+f_{R}^{⊥}$ and $-f_{R}^{⊥}$ , with the same magnitude of ℓΩ . With the carrier wave frequency of LDE shift, the total frequency shift is still symmetrically distributed with LDE frequency shift as the center. Such that, it is reasonable to take the mean value of the measured frequency as the LDE frequency shift. [See Supplement 1 for the detailed obtaining method of LDE shift.]

The frequency shift introduced by the vibration comes from the performance of the translation stage itself which is inevitable. However, the second frequency shift is introduced by the superposition optical vortex can be controlled and further investigated. In all Doppler velocimetry, it is usually a tough work to identify the direction of the motion. In our measurement, we take the conjugated optical vortex as the detection beam to illuminate the object, therefore arousing the conjugated rotational frequency shift with the same magnitude. Upon this condition, the rotational direction information is missing in the measurement. However, the generation of the superposition optical vortex with both different topological charges and opposite signs is convenient by existing technology. If the superposition optical vortices with opposite signs and different topological (suppose $\left|\ell_{1}\right|>\left|\ell_{2}\right|$ ) are employed in this scheme, the composite frequency shifts would be $f_{L}^{∥}+f_{R}^{⊥}\left(\ell_{1}\right)$ and $f_{L}^{∥}-f_{R}^{⊥}\left(\ell_{2}\right)$ . Then the mean value of the frequency shift is not the LDE shift anymore but becomes $f_{L}^{∥}\pm \Delta f_{R}^{⊥}/2$ associate with the rotational direction of the object, where $\Delta f_{R}^{⊥}=f_{R}^{⊥}\left(\ell_{1}\right)-f_{R}^{⊥}\left(\ell_{2}\right)$ . By comparing with the measurement results of the conjugated optical vortex as the detection beam, the rotational direction can be recognized conveniently.

## 4 Conclusions

In summary, confront with the problem of target compound motion measurement, we proposed a double interference method to realize the separate measurement of rotation and rectilinear motion. The interference properties between different kinds of beams, and the phase characteristics after interacting with complex motion, are fundamental to the interpretation of experiments used in measuring the speed of the object. Three kinds of interference measurement methods based on optical vortex are analyzed. The interference measurement between plane wave and single-mode optical vortex is only sensitive to the rectilinear motion and immune to the rotation, compared with the self-interference measurement is sensitive to the rotation but immune to the linear motion. Through the combination of self-interference of the optical vortex and the reference interference with the plane wave, the rotational and rectilinear speed can be achieved dependently and simultaneously. Moreover, if the conjugated state of the superposition optical vortex is changed, it shows a potential ability to recognize the rotational direction of the object. This work may provide a new way in the detection of free space moving object and stimulate the invention of new optical detection equipment.

## Supplementary Material

Supplementary Material
