# Supplementary material for: Compound motion detection based on OAM interferometry
Source: Nanophotonics. 2022 Feb 15;11(6):1127–35. doi: 10.1515/nanoph-2021-0622 (PMC11502107; doi:10.1515/nanoph-2021-0622)
Supplement: Supplementary file 1 — Supplementary Material [file j_nanoph-2021-0622_suppl.docx]

Compound motion detection based on OAM interferometry: supplemental document

**This Document includes:**

- Materials and Methods
- Figures.S1 and S2.
- Table S1

**Frequency shift introduced by conjugated OAM beams and plane wave**

Based on the coherent superposition principle of light, the electric field of three coherent beams converge at one point can be expressed as,

$$E^{2}=E_{1}^{2}+E_{2}^{2}+E_{3}^{2}+2E_{1}E_{2}+2E_{1}E_{3}+2E_{2}E_{3}$$

In our experiment, the electric field of three interference beams are,

where for the conjugated OAM beams denotes the topological charge. The polarization state of these three beams is all the linear polarization and $\sigma$ can take the value of 0. Combine Eqs. , ,and with Eq., and leaves the scaler intensity of the interference field we can obtain,

where $\text{I}_{n}(n=1,2,3)$ is the scaler field intensity of the three beams. and $\Delta z$ denotes the azimuthal phase difference and light path difference of the interference beams, respectively.

If implement the above interference scheme into a motion detection frame, then the variation of the scaler intensity can be determined associated with the speed of the object. To realize the measurement of the compound motion, we use the conjugated superposition optical vortices $\text{E}_{OAM}^{1}+\text{E}_{OAM}^{2}$ as the detection beam and then interference with the plane wave $\text{E}_{P}^{3}$. After interaction with the moving target, the phase change of the scattered light can be expressed by azimuthal change and longitude light path change $\Delta z\text{=v}_{0}t$. The scaler intensity of the interference beam therefore can be written as,

It is obvious from the above formula that the scaler intensity of the interference field is decided by three factors. And the principle of the intensity change over time can be obtained by $\partial I(t)/\partial\text{t}$. Implementing derivation on Eq. we can obtain three modulated frequency components,

where the first two frequencies are the compound frequency shift of the LDE and RDE, and the third one is the RDE frequency shift.

**Obtaining the LDE frequency by STFT**

Limited by the character of the translation stage, the vibration of the platform is inevitable in the experiment. The linear Doppler frequency is affected and fluctuating correspondingly. It’s reasonable to take the center value to represent the linear Doppler frequency shift. The signal recorded by the photodetector is $x(t)$, with the sampling frequency being $F$, and the number of samples being $N$. We first conduct the short time Fourier transformation on the original data, the STFT diagram can be calculated by $STFT(t,f)=\int_{-\infty}^{+\infty} x(\tau)h(\tau-t)e^{-j2\pi ft}d\tau$. Secondly, a matched threshold-value filtering algorithm is implemented on the STFT results. And then take the frequency with the maximum strength at all times to represent the compound frequency shift. In this process, the 3D STFT diagram becomes the 2D frequency-time varying diagram. Finally, the frequency-time variation result is best fitted with sinusoidal function. From the fitting result, the value of the LDE frequency shift can be directly recognized from the fitting equation. Taking the one experiment with the linear speed of $v=5.5mm/s$ as an example, the whole process is shown in Fig. S1.

**Fig. S1**


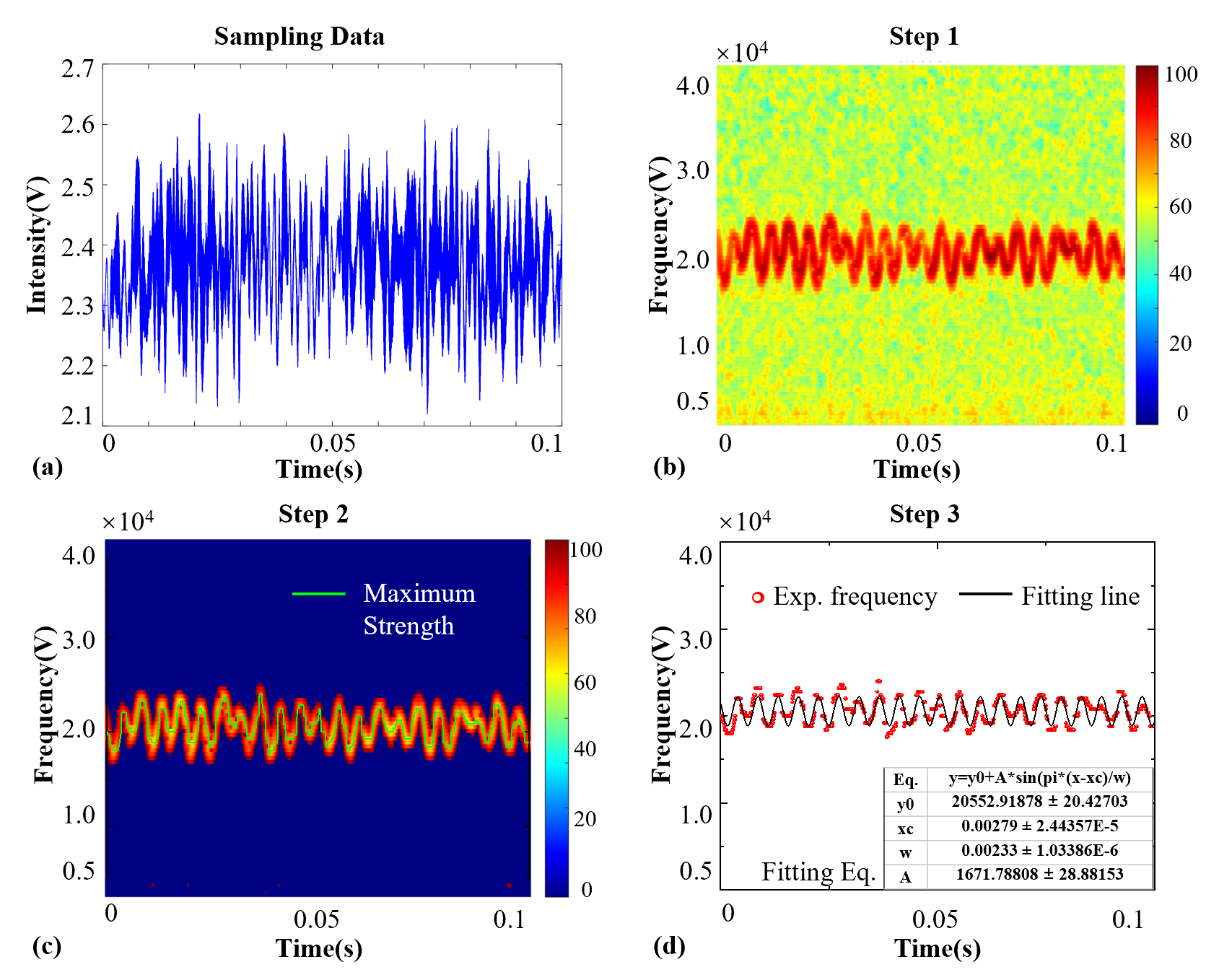


Fig. S The LDE frequency acquisition process. (a) present the original data in time domain. (b) shows the STFT results. (c) is the result after threshold-value filtering of STFT results. (d) Fitting the frequency signal by sinusoidal function. The center value $y0$ of the fitting equation is taken as the LDE frequency shift.

**Fig. S2**


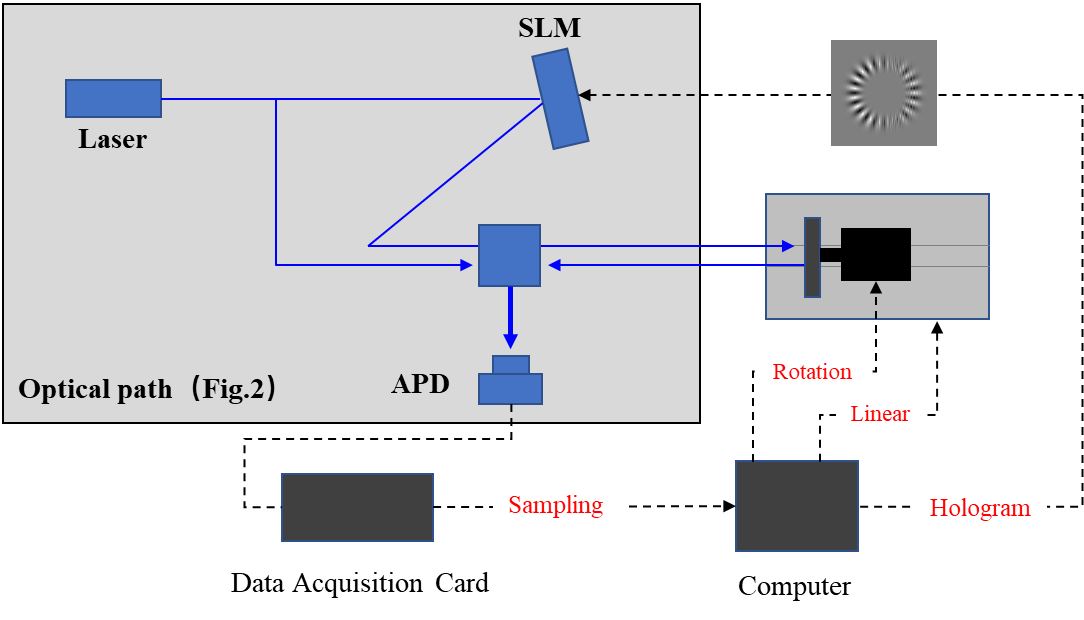


Fig. S The full detection scheme including optical path and the electric part. Computer play the role as the controller to control the motion speed and the generation of the OAM beam. The intensity of the interference is captured by APD and then sampling by DAC.

**Table. S1**

| Group | 1 | 2 | 3 | 4 | 5 | 6 | 7 | 8 | 9 |
| --- | --- | --- | --- | --- | --- | --- | --- | --- | --- |
| Rotation Speed  (rps) | 2.78 | 3.06 | 3.33 | 3.61 | 3.89 | 4.17 | 4.44 | 4.72 | 5.00 |
| *f*_RDE_(Hz) | 114.4 | 120.5 | 132.8 | 145 | 155.6 | 166.3 | 178.5 | 189.2 | 199.9 |
| Rectilinear Speed  (mm/s) | 1.5 | 2.0 | 2.5 | 3.0 | 3.5 | 4.0 | 4.5 | 5.0 | 5.5 |
| *f*_LDE_(Hz) | 5662 | 7429 | 9328 | 11600 | 14200 | 14820 | 16870 | 18830 | 20710 |

Tab. S The measured signal frequency upon different rotational and rectilinear speed. The $f_{RDE}$ is obtained by Gaussian fitting in the low frequency domain and $f_{LDE}$ is obtained through the method presented in Fig. S2. The corresponding velocity and the relative error are presented in Fig.5.
